# Supplementary material for: Unorthodox PCNA Binding by Chromatin Assembly Factor 1
Source: Int J Mol Sci. 2022 Sep 21;23(19):11099. doi: 10.3390/ijms231911099 (PMC9570017; doi:10.3390/ijms231911099)
Supplement: Supplementary file 1 [file ijms-23-11099-s001.zip › ijms-1862444-supplementary.pdf]

Supplementary Materials-Expression and purification of recombinant proteins

S1. Expression and purification of GST-PIP fusion proteins in Escherichia coli

Wild-type p150 PIP and R426D peptides were constructed as GST-fusion proteins with a tyrosine at the C-terminus of the peptide. The C-terminal tyrosine was added to aid in peptide detection during the purification by measuring the absorbance at 280 nm. The DNA fragments encoding the GST fusion proteins were inserted into a pGEX-TEV vector, which is essentially a modified pGEX-2T vector where the thrombin cleavage site was replaced with a TEV cleavage site [63]. The pGEX-TEV plasmid was digested with *Bam*HI and *Eco*RI and ligated to DNA fragments encoding either the p150 PIP wild-type and R426D. The plasmids pGEX-TEV-PIP wild type and pGEX-TEV-PIP R426D were confirmed by DNA sequencing and then expressed in *Escherichia coli* host strain TOPP2 (Stratagene) with 100 µg/ml ampicillin to saturation at 37°C. The next day, the saturated cells were diluted to 0.05 OD measured at A_{600} nm and the cells were grown at 37°C until the cells reach the mid-exponential phase of 0.6-0.8 at OD A_{600} nm. The cells were then induced with 1 mM isopropyl-D-thiogalactopyranoside (IPTG) for 4 h. Eight litres of cell culture were grown each for the wild-type and R426D mutant. The cells were then harvested by centrifugation in an Avanti J-26XP high-performance centrifuge from Beckman Coulter with a fixed angle rotor (JLA-8.1000) at 9000 x g for 30 min at 4°C. The pellet from 1 litre cells equivalent was lysed in 10 ml of lysis buffer containing 20 mM Tris pH 7.5, 1 M NaCl, 0.2 mM EDTA, 1 mM DTT, 50 µg/ml lysozyme, 1 mM AEBSF and one tablet of cOmplete™ Mini EDTA-free Protease Inhibitor Cocktail Tablets (Roche). The cell pellet was sonicated in the lysis buffer on ice, at power output level 4 with 10 seconds pulse on and 30 seconds off at 6 watts using a Misonix 3000 Ultrasonic cell disruptor and wider probe. The lysate was clarified by centrifugation for 30 min in an Avanti J-26XP with a fixed angle rotor 25.50 at 30966 x g at 4°C. The clarified supernatant was incubated on a nutator for 6 h at 4°C with 10 ml of glutathione-Sepharose (GSH) resin (Amersham) prewashed and equilibrated with lysis buffer. After incubation the GSH resin was pelleted, the unbound supernatant was removed, and the resin was washed thrice with lysis buffer and twice with Tobacco Etch Virus (TEV) protease buffer (20 mM sodium phosphate pH 6.5, 50 mM NaCl, 2 mM DTT). The peptide was cleaved from the GSH resin by incubating in the presence of TEV protease overnight at 4°C. The next day, the supernatant was harvested by centrifugation, and the GSH resin was washed twice with 20 mM sodium phosphate pH 6.5 buffer. The fractions were run on a 15% polyacrylamide gel to track the purification and TEV cleavage efficiency. The gel was stained with Coomassie brilliant blue G-250 with 50% (v/v) methanol, 10% (v/v) glacial acetic acid and destained with distilled water with 0.1% (v/v) acetic acid. The supernatant after GSH purification and TEV cleavage contains the purified peptide, and this peptide was further purified to remove the TEV enzyme on an SP-Sepharose (GE Healthcare) ion-exchange chromatography column. After elution from SP-Sepharose columns, pure fractions of the peptide were dialyzed against 4 litres of water overnight using a 1 kDa MWCO dialysis membrane. The dialyzed pure peptide was further quantified, lyophilized, and stored at -80°C until used for ITC experiments.

The protocol had to be modified to purify the mutant peptide of pGEX-TEV-PIP R426D. Even though the mutant protein was expressed at a similar level as the wild-type, the mutant peptide pGEX-TEV-PIP R426D after GSH purification did not bind efficiently to the SP-Sepharose column. This problem was circumvented by modifying the protocol for pGEX-TEV-PIP R426D by carrying out the TEV cleavage followed by performing an additional gel filtration step (gel filtration buffer 20 mM Tris pH 7.5, 150 mM NaCl, 1 mM DTT) using HiLoad 26/600 Superdex™ 75 pg column instead of SP-sepharose ion exchange. The eluted peak fractions corresponding to pGEX-TEV-PIP R426D from gel filtration were pooled and lyophilized and stored at -80°C until used for ITC experiments.

S2. Expression and purification of p150L in Escherichia coli

A DNA fragment encoding human p150 residues 342-475 was cloned into the pET28a vector (kanamycin resistant) in-frame with a C-terminal 6X-histidine tag. For p150L expression, this plasmid was transformed into *Escherichia coli* BL21(DE3) RIL cells (Stratagene). Single colonies were inoculated and grown overnight to saturation at 37°C in 2X YT medium containing 50 µg/ml kanamycin. The next day, cells were diluted and grown to mid-exponential phase (A_{600nm} = 0.6 - 0.8) at 37°C and protein expression was induced at 20°C with 0.5 mM isopropyl-β-D-thiogalactopyranoside (IPTG) for 16 h.

The cells were harvested in an Avanti J-26XP centrifuge from Beckman Coulter with a fixed angle rotor (JLA-8.1000) at 9000 x g for 30 min at 4°C. The pellets from 1L of cell culture were resuspended in 50 mL of lysis buffer containing 50 mM sodium phosphate pH 7.2, 2.5 M NaCl, 75 mM imidazole, 100 µg/mL lysozyme, 1 mM AEBSF, one tablet of cOmplete Mini EDTA-free Protease Inhibitor Cocktail (Roche) and 0.1 mM TCEP. The cells were lysed in a cold room on a nutator for 30 min, followed by sonication at power output level 4 with 10-second pulses on and 30-second off at 6 watts using a Misonix 3000 Ultrasonic cell disruptor and their wider probe. The lysate was clarified by centrifugation for 30 min in an Avanti J-26XP with a fixed angle rotor 25.50 at 30966 x g at 4°C. The supernatant was incubated overnight at 4°C with nickel-nitrilotriacetic acid agarose beads (Qiagen) that were pre-equilibrated in the lysis buffer. After incubation with the beads, the supernatant was removed after centrifugation and the beads were washed thrice in wash buffer containing 50 mM sodium phosphate pH 7.2, 2.5 M NaCl, 75 mM imidazole, and 0.1 mM TCEP. The p150L protein was eluted with a buffer containing 50 mM sodium phosphate pH 7.2, 2.5 M NaCl, 500 mM imidazole, and 0.1 mM TCEP. After elution, p150L-His was concentrated using a 3 kDa MWCO Amicon concentrator. The presence of 2.5 M NaCl was to prevent the p150L protein, which is rich in K/E/R residues, from co-purifying with non-specifically bound nucleic acids. This method was further refined by performing benzonase treatment (90 units/mL), followed by three 15 minutes 1M NaCl washes followed by dialysis were required to remove nucleic acids that got co-purified as a contaminant. After concentration, 500µl to 1ml of p150L protein was dialysed in a 7 kDa MWCO Snakeskin dialysis membrane (Thermo Fisher Scientific) in 4 litres of dialysis buffer compatible with biophysics experiments for 4 h, repeated thrice. The protein was harvested the next day and the protein sample was stored at -80°C until used. The concentration of p150L was estimated by measuring A_{205nm} (p150L is devoid of Trp or Tyr residues).

For NMR experiments, CAF-1 p150L was isotopically labeled by growing *Escherichia coli* in M9 minimal medium containing ^{15}N-ammonium chloride (Sigma) and/or ^{13}C-glucose (Sigma) as the sole nitrogen and carbon sources. A 10X ^{15}N minimal medium (1 litre) was prepared by dissolving 130 g KH_2PO_4, 100 g K_2HPO_4, 90 g Na_2HPO4, and 24 g K_2SO_4. The pH was adjusted to 7.2 with NaOH before adding 10 g of ^{15}N-NH_4Cl. The 200X trace elements stock solution (1 litre) was prepared by dissolving 60 g $CaCl_2.2H_20$, 60 g $FeSO_4.7H_20$, 115 g $MnCl_2.4H_20$, 0.8 g $CoCl_2.6H_20$, 0.7 g $MnSO_4.7H_20$, 0.3 g $CuCl_2.2H_20$, 0.02 g H_3BO_3, 0.25 g $(NH_4)_6Mo_7O_{24}.4H_20$, 50 g EDTA.2NaCl in 750 ml H_2O and once dissolved the volume was made up to 1L, which was sterilized through a 0.2 micron filter. Regular glucose or ^{13}C glucose was made as 8% (w/v) 40X stock and filter sterilized. Twenty-five millilitres of this solution were used per litre of culture medium. Five grams of yeast extract was dissolved in 50 mL ddH_20 (10% w/v final) and was autoclaved. To make the 1X (1L) minimal medium, 10X ^{15}N solution was diluted with ddH_20 and added with 25 mL 40X glucose, 5 mL 1M $MgCl_2$, 5 mL 200X trace elements, 100 µl 10% (w/v) yeast extract, 1 mL of antibiotic (kanamycin; 1000X stock was 50 mg/mL). The cells were grown as an overnight 50 mL primary culture, which was used to inoculate 1 litre of M9 medium, and induction with IPTG was performed as mentioned above for the rich media.

S3. Expression and purification of wild-type and mutant PCNA proteins in Escherichia coli

PCNA wild-type and mutants were expressed and purified as previously reported [18]. In short, wild-type or mutant plasmids in pET23-C-His6-PCNA vectors were transformed and expressed in Codon Plus BL21(DE3) pLysS *E. coli* (Stratagene) cells. Single colonies were inoculated and grown overnight in the Luria-Bertani medium containing 100 μg/mL ampicillin to saturation at 37°C. The next day the cells were diluted and grown to mid-exponential phase (0.6-0.8) at $OD_{600\ nm}$, and the expression was induced with 1 mM isopropyl-β-D-thiogalactopyranoside (IPTG) for 4 h. PCNA mutants were transformed and expressed in a similar way.

Cells were spun in an Avanti J-26XP high performance centrifuge from Beckman Coulter with a fixed angle rotor (JLA-8.1000) at 9000 x g for 30 min at 4°C and the pellets from 1 litre of cell culture were resuspended in 50 mL of lysis buffer containing 50 mM sodium phosphate pH 8.0, 150 mM NaCl, 10% glycerol, 20 mM imidazole, 0.02% nonidet P-40, 2 mM DTT. The cell pellet was sonicated at power output level 4 with 10 seconds pulse on and 30 seconds off at 6 watts using a Misonix 3000 Ultrasonic cell disruptor and wider probe in the lysis buffer. The lysate was clarified by centrifugation for 30 min in an Avanti J-26XP with a fixed angle rotor 25.50 at 30966 x g at 4°C. The supernatant was incubated overnight at 4°C with pre-equilibrated nickel-nitrilotriacetic acid agarose beads (catalogue number - 30230, Qiagen) in the lysis buffer. After incubation, the beads were washed three times with wash buffer and PCNA wild-type or mutants were eluted with an elution buffer containing 500 mM imidazole.

The eluate was subsequently purified with a Superdex™ 200 gel filtration column (GE Healthcare) in gel filtration buffer 20 mM Tris pH 7.5, 150 mM NaCl, 1 mM DTT. The fractions corresponding to PCNA homotrimer were pooled, concentrated with an amicon concentrator with 3 kDa MWCO, quantified using absorbance at $A_{280\ nm}$, and stored at -80°C until used.

S4. Human PCNA expression and purification for crystallography

pT7-hsPCNA plasmid (kindly donated by Prof. Malcolm Walkinshaw, University of Edinburgh) was introduced into chemically competent *E. coli* strains, Overexpress C43 (DE3) cells (Lucigen), which were grown overnight on selective agar plates with 100 μg/ml ampicillin. Transformed C43 (DE3) cells were grown in 3 ml 2X YT (100 μg/ml carbenicillin) pre-cultures to OD_{600} > 0.6. These were added to 450 ml 2X YT (100 μg/ml carbenicillin), cells were grown by shaking incubation (37°C, 250 rpm) until an OD_{600} nm of 0.6-0.8. Cells were induced with 1 mM IPTG and incubated at 37°C for 3 h. Cells were harvested by centrifugation (4000 x g, 30 min, 4°C) and resuspended in 45 mL Sepharose Buffer-A (15.5 mM 1-methyl-piperazine, 15.5 mM Bis-Tris, 7.8 mM Tris-buffer pH 8.5 containing one tablet of cOmplete Mini EDTA-free Protease Inhibitor Cocktail (Roche). Cells were lysed by multiple passes through an Emulsiflex C5 instrument (Avestin), at variable pressure. Successful PCNA purification was achieved by manual correction of stroke pressure to < 750 kPsi. The lysate was clarified by centrifugation (48000 x g, 45 min, 4°C). The supernatant was applied to two 1 ml HiTrap Q FF (GE Healthcare) connected in series, pre-equilibrated in Buffer-A (15.5 mM 1-methyl-piperazine, 15.5 mM Bis-Tris, 7.8 mM Tris-buffer pH 8.5) on an ÄKTA Explorer chromatography system (GE Healthcare). After loading, the column was washed with 30 ml 20% Buffer-B (15.5 mM 1-methyl-piperazine, 15.5 mM Bis-Tris, 7.8 mM Tris-buffer pH 8.5, 1M NaCl), followed by a 20 ml gradient of 20-57% Buffer-B and a 10 ml gradient of 57-100% Buffer-B. Relevant fractions (as judged by SDS-PAGE) were concentrated to 2.5 mL using a Vivaspin20 centrifugal concentrator (GE Healthcare) before loading for buffer exchange on a PD-10 Desalting Column (GE Life Sciences) pre-equilibrated in Buffer-C (5.1 mM Na_2HPO_4, 5.1 mM Sodium formate, 10.2 mM Sodium acetate, pH 5.5). Fractions were loaded onto a 5 mL HiTrap Heparin HP

column (GE Healthcare) pre-equilibrated in Buffer-C. The column was washed with 25 mL 15% Buffer-D (5.1 mM Na_2HPO_4, 5.1 mM Sodium formate, 10.2 mM Sodium acetate pH 5.5, 1M NaCl), followed by a 50 mL gradient of 15-100% Buffer-D. Relevant fractions were pooled and concentrated using a Vivaspin20 centrifugal concentrator to 400 μL. This was split into 2 x 200 μL each, injected separately onto a Superdex™ 200 gel filtration column (GE Healthcare) pre-equilibrated in Buffer-E (25 mM Tris-buffer, 25 mM NaCl, 0.5 mM EDTA, 10% glycerol, pH 7.5) at 1.5 mL/min. Relevant fractions were pooled and concentrated to ~10 mg/mL before use in crystallisation trials.

Supplementary Table

**Sample details**		
Protein name		Chromatin assembly factor 1 subunit A
Short name		CAF-1 p150
Organism		*Homo sapiens*
Source		recombinant, from *E. Coli*
Accession number		Q13111
Amino acids range		342-475
Extinction coefficient at 205nm	$M^{-1}cm^{-1}$	489260 M-1 cm-1
Oligomeric state		probably dimer
Monomer molecular weight, including tags	kDa	17
**SEC-SAXS data collection parameters**		
Chromatography		
Instrumentation		GE Healthcare ÄKTAmicro
column		Superdex 200 Increase 10/300
loading concentration	mg/ml	5.8
injection volume	µl	500
flow rate	µl/min	50
solvent		20 mM phosphate pH 7.0, 200 mM NaCl, 0.1 mM TCEP
X-ray scattering		
Instrumentation		Xenocs BioXolver L
X-ray source		Excillum MetalJet D2+ 70 kV (95% gallium)
Wavelength	Å	1.34
Beam size	µm	80 × 20
Detector		Dectris PILATUS3 R 300K
Detector distance	mm	600
q measurement range	$Å^{-1}$	0.0134 – 0.5778
Exposure		Continuous 60 s data-frame measurements
Sample temperature	°C	20.0
**Software employed for SAXS data reduction, analysis and interpretation**		
SAXS data reduction		RAW 2.0.3
Basic analysis (Guinier, P(r), Vp)		RAW 2.0.3, SAXSMoW 2.1
Shape/bead modelling		DAMMIF (ATSAS 3.0.1)
Electron density modelling		DENSS 1.6.2
3D graphic model representation		PyMOL 2.1.0
**Structural parameters**		
Guinier analysis		
I(0)	cm^{-1}	$39 \pm 2 \cdot 10^{-4}$
R_g	Å	40.8 ± 2.5
q-range	$Å^{-1}$	0.0134 – 0.03208
qR_g max		1.31
R^2		0.9403
Molecular weight (Piiadov 2019)	kDa	34.8
P(r) analysis (GNOM)		
I(0)	cm^{-1}	0.0041 ± 0.0002
R_g	Å	47.1 ± 2.1
D_{max}	Å	172
q range	$Å^{-1}$	0.0134 – 0.2497
2		0.9433
Total estimate		0.6308
**Shape model-fitting results**		
DAMMIF (default parameters, 20 calculations)		
q range for fitting	$Å^{-1}$	0.0134 – 0.3056
symmetry, anisotropy assumptions		P2, none
NSD (standard deviation)		0.883 ± 0.032
$\chi 2$ range		0.932 – 0.942
resolution	Å	34 ± 3
number of rejections (NSD > mean NSD + 2SD)		1
DAMMIN refinement		
q range for fitting	$Å^{-1}$	0.0134 – 0.3056
symmetry, anisotropy assumptions		P1, none
$\chi 2$		1.049
Rg	Å	42.3
Dmax	Å	140.5
Excluded volume	$Å^3$	23512

Supplementary Table S1. Data collection parameters for the SEC-SAXS experiment. Scattering data has been deposited in the Small Angle Scattering Biological Data Bank (SASBDB) under the accession number SASDP79.

Supplementary Figures

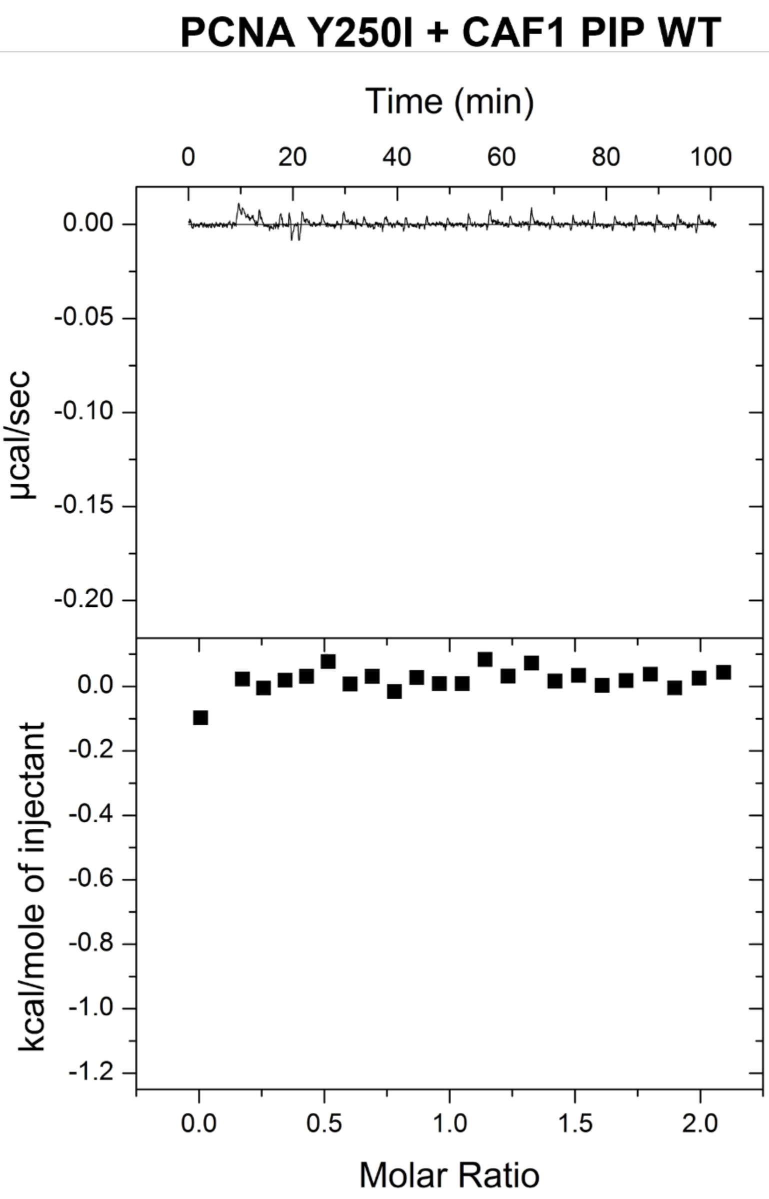

Supplementary Figure S1. In ITC experiments, the CAF-1 PIP wildtype peptide was progressively titrated into a sample cell containing PCNA Y250I. The upper half of each panel shows the measured heat exchanges following each peptide injection. The lower half of each panel shows the enthalpic changes as a function of the molar ratio of peptide to PCNA monomer. The black squares correspond to individual injections. Titrations were performed in 10 mM sodium phosphate, pH 7.0, 10 mM NaCl and at 23 ^{0}C

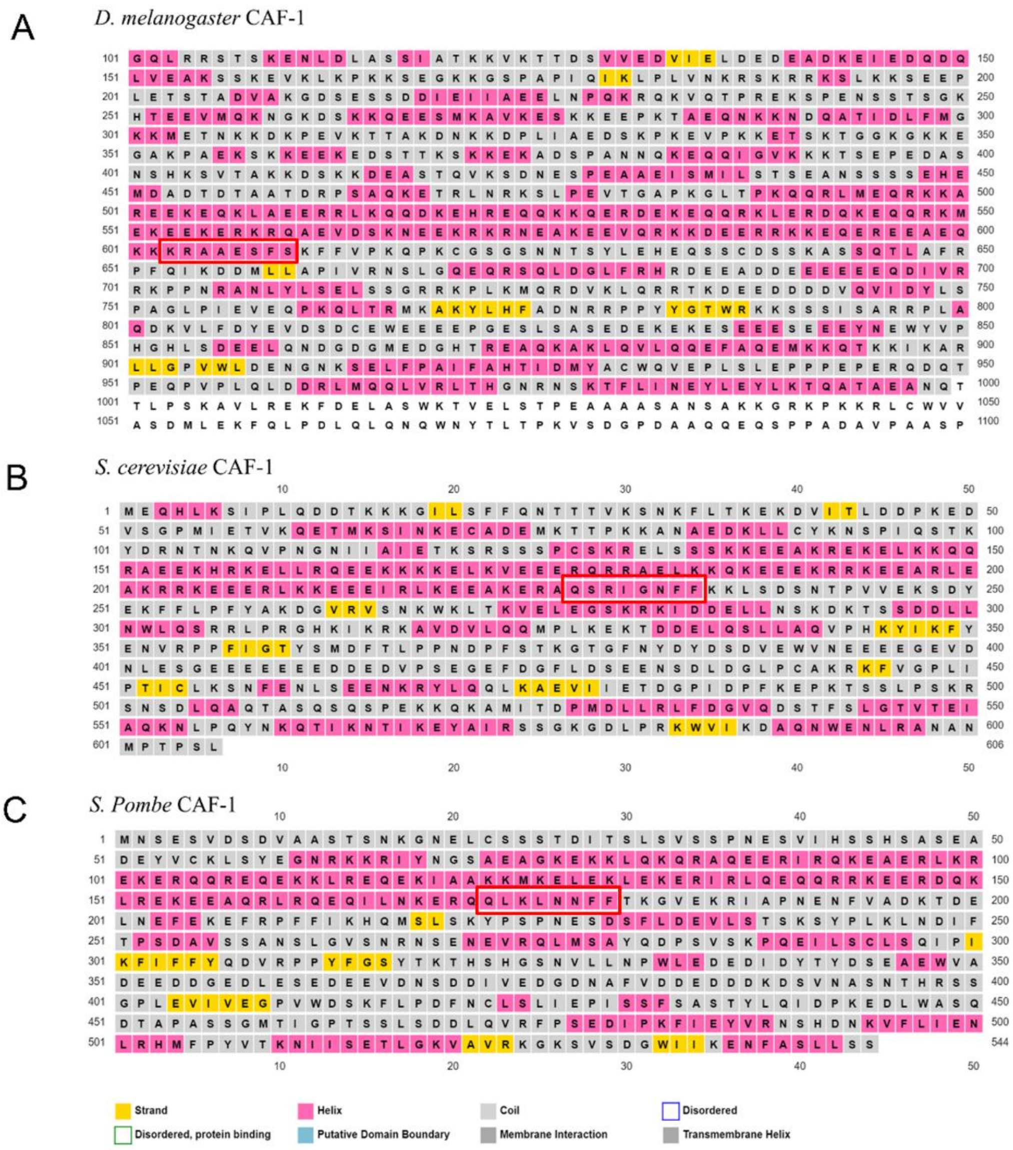

Supplementary Figure S2. Secondary structure prediction analyses of CAF-1 p150 homologues.

Even though *D. melanogaster*, *S. cerevisiae* and *S. pombe* are evolutionarily distant from humans, the large fragment encoding the KER domain of p150 homologues is predicted to form an unusually long α-helix (100-120 amino acids depending on species) that ends abruptly in the PIP residues. It is important to note that in both species of yeast, CAF-1 PIP sequences are canonical.

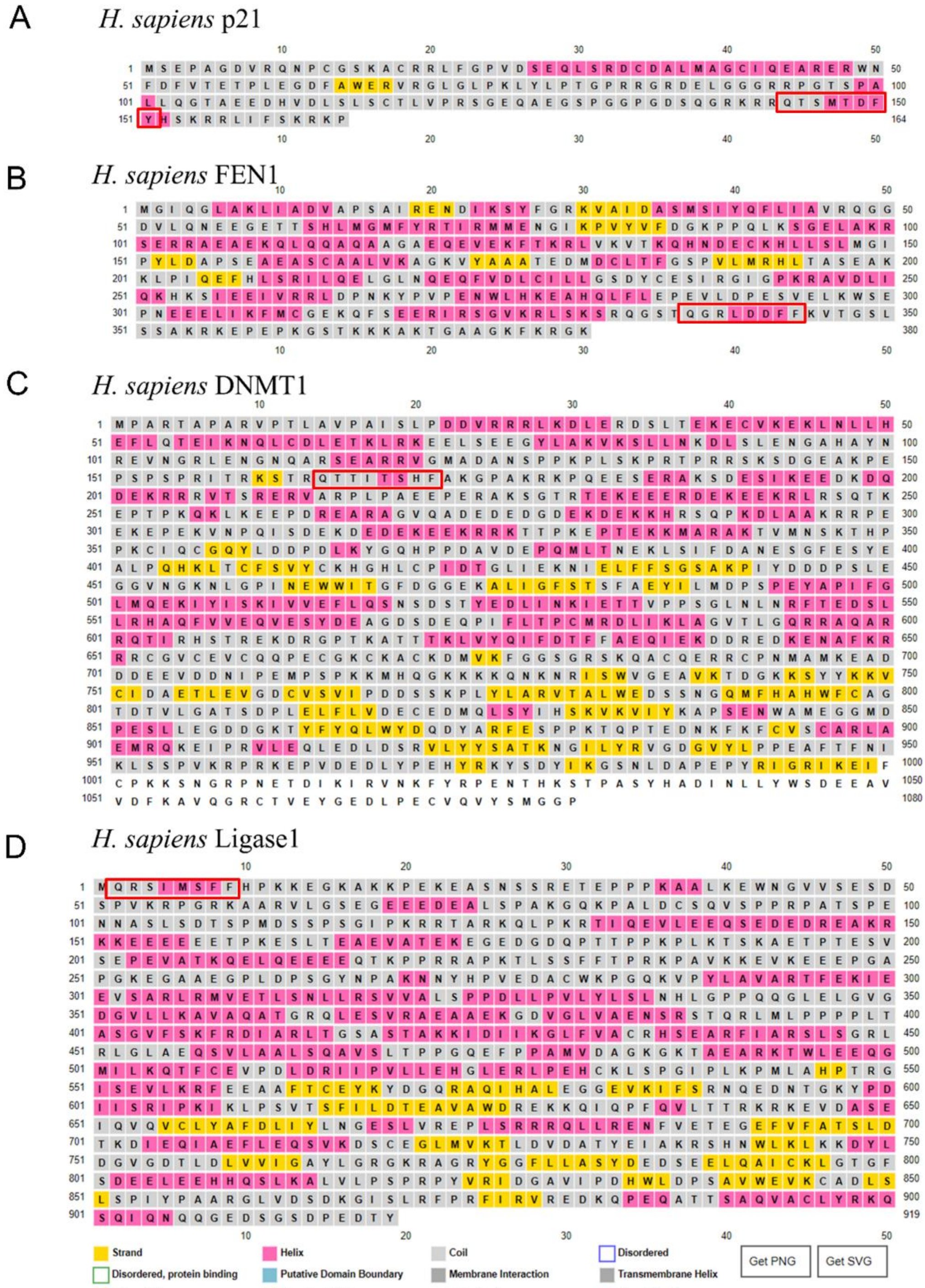

Supplementary Figure S3. Secondary structure prediction analyses of replication enzymes

Secondary structure prediction analyses of several DNA replication enzymes that contain canonical PIPs. None of the proteins (p21, FEN1, DNMT1 and DNA ligase I) are predicted to contain a long α-helix that ends with a PIP, unlike human CAF-1 p150 and its homologues.

A *H. sapiens* pol kappa

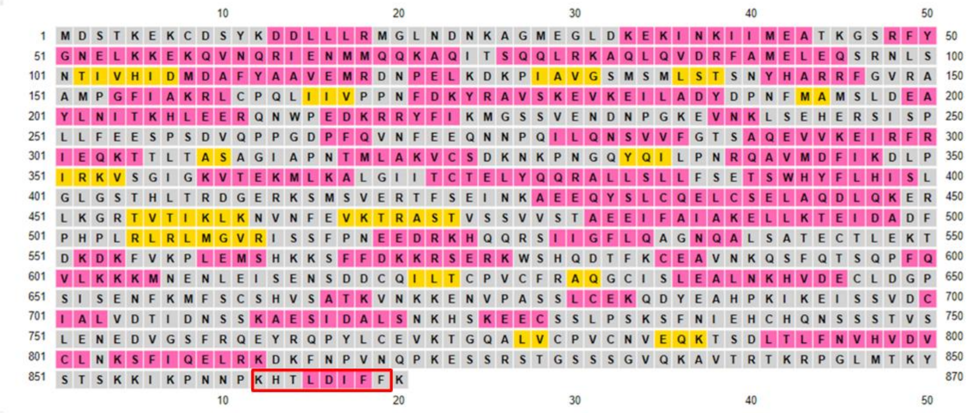

B *H. sapiens* pol iota

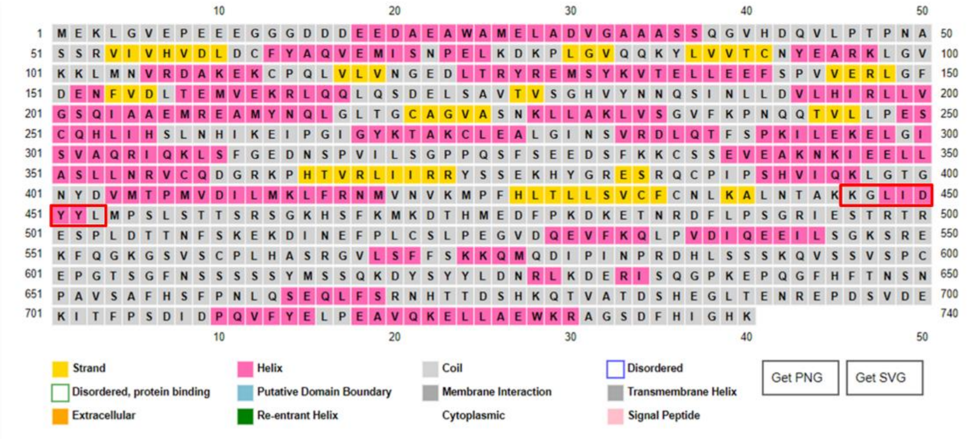

Supplementary Figure S4. Secondary structure prediction analyses of non-canonical error prone polymerases Pol kappa and Pol iota. They are not predicted to contain a long α-helix that ends with a non-canonical PIP, unlike human CAF-1 p150 and its homologues.

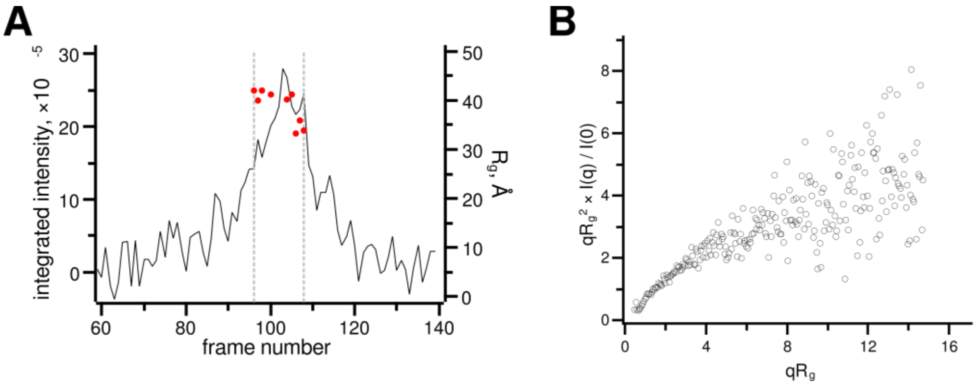

Supplementary Figure S5. (A) SAXS frame integrated intensity (buffer subtracted, baseline corrected) plotted against frame number (each frame is a 60 sec exposure from the elution at 0.05 mL/min). Red dots correspond to the calculated radius of gyration (Rg) for the corresponding frame. Portion between the dotted grey lines was used for averaging and further processing. (B) Dimensionless Kratky plot representation of the experimental data.

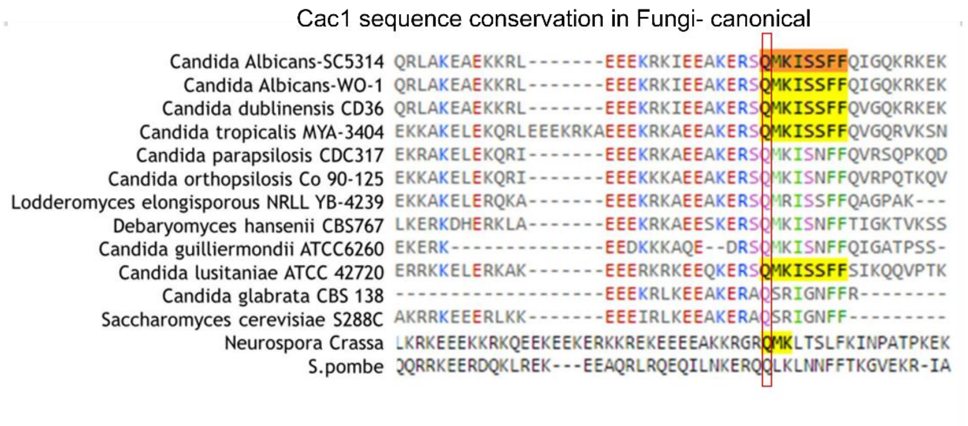

Supplementary Figure S6. Cac1 sequence in various fungal species are canonical.
